# The San Antonio Meeting, Texas Medical Association

**Published:** 1889-04

**Authors:** 


### EDITORIAL DEPARTMENT.

F. E. DANIEL, M. D., EDITOR AND PUBLISHER.

THIS JOURNAL, by unanimous vote of the members, was made the Official Organ of the Austin District Medical Society.

COLLABORATORS:

*Dr. R. M. Swearingen, Austin.*
*Prof. B. E. Hadra, M. D., Galveston.*
*Prof. Geo. Cupples, M. D., San Antonio.*
*Dr. T. C. Osborn, Cleburne, Texas.*
*Dr E. J. Doering, Chicago.*
*Dr. E. J. Beall, Fort Worth.*
*Dr Odo Betz, Germany.*
*Dr. J. W. McLaughlin, Austin.*
*Dr. T. J. Tyner, Austin.*
*Prof. J. F. Y. Paine, M. D., Galveston.*
*Dr. R. H L. Bibb, Mexico.*
*Dr. T. J. Bennett, Austin.*
*Dr. Bat Smith, Wharton.*
*Dr. E. Meierhof, New York.*
*Prof. W. B. Rogers, M. D., Memphis, Tenn.*

### THE SAN ANTONIO MEETING, TEXAS MEDICAL ASSOCIATION.

Within a very few days after this issue of the JOURNAL reaches its readers, the Texas State Medical Association will assemble in its 21st annual session.—The meeting will be held at San Antonio, beginning Tuesday morning, April 23rd, and will continue four days.

Those physicians who expect to attend for the purpose of becoming members, are here reminded of the necessity of bringing with them proper credentials, either a diploma, or a certificate from the clerk of the County or District Court in the County in which they reside, that the diploma has been duly registered. The payment of the initiation fee and one years dues—in all $10, is also required.

We beg also to remind delinquent members of a resolution which was passed at the Dallas meeting, which virtually wipes out all arrears; and a member who owes two, three or more year's dues, can be reinstated by paying the initiation fee and one year's dues, $10, as required of new members.

The Secretary also begs to repeat the request that all papers to be read by members—either in open session, or in sections, should be carefully prepared—written in ink or type written, and duly paged. Proper names and technical terms *must* be plainly written. They should also be endorsed on the back with name o author and subject, and the section to which it belongs. After being read, the author should *see* that his paper is placed in the hands of the general Secretary. The cbservance of the above will save much trouble and annoyance, when it comes to editing papers for publication in the transactions.

Secretaries of sections are also respectfully urged to take full notes of all important discussions, and at the close of the meeting, hand to the Secretary all papers read in their respective sections, together with notes of proceedings had, and the discussion on papers, in the order in which they occurred.

On arrival, delegates from local societies are respectfully urged to furnish the Secretary with a list of their representatives present, and the officers of their respective societies. The co-operation of all, is respectfully solicited, toward securing a perfect record of the proceedings.

